# Isolation and characterization of NBS–LRR resistance gene analogues from mango

**DOI:** 10.1080/13102818.2014.931706

**Published:** 2014-07-08

**Authors:** Xintao Lei, Quansheng Yao, Xuerong Xu, Yang Liu

**Affiliations:** ^a^ Chinese Academy of Tropical Agriculture Science, South Subtropical Crops Research Institute, Zhanjiang, Guangdong, P.R. China; ^b^ Chinese Academy of Tropical Agriculture Science, Zhanjiang Experiment Station, Zhanjiang, Guangdong, P.R. China

**Keywords:** nucleotide-binding site (NBS), leucine-rich repeat (LRR), resistance gene, mango, diversity

## Abstract

The nucleotide-binding site (NBS)–leucine-rich repeat (LRR) gene family is a class of *R* genes in plants. NBS genes play a very important role in disease defence. To further study the variation and homology of mango NBS–LRR genes, 16 resistance gene analogues (RGAs) (GenBank accession number HM446507-22) were isolated from the polymerase chain reaction fragments and sequenced by using two degenerate primer sets. The total nucleotide diversity index *Pi* was 0.362, and 236 variation sites were found among 16 RGAs. The degree of homology between the RGAs varied from 44.4% to 98.5%. Sixteen RGAs could be translated into amino sequences. The high level of this homology in the protein sequences of the P-loop and kinase-2 of the NBS domain between the RGAs isolated in this study and previously characterized *R* genes indicated that these cloned sequences belonged to the NBS–LRR gene family. Moreover, these 16 RGAs could be classified into the non-TIR–NBS–LRR gene family because only tryptophan (W) could be claimed as the final residual of the kinase-2 domain of all RGAs isolated here. From our results, we concluded that our mango NBS–LRR genes possessed a high level of variation from the mango genome, which may allow mango to recognize many different pathogenic virulence factors.

## Introduction

Plant diseases are among the many major factors which limit the yield of crops; fungal, bacterial and viral pathogens, which are ubiquitous, are able to infect almost all species. Many resistance (*R*) genes are single genotype locus genes that confer resistance against one pathogen.[1] *R* genes are known to contain two conserved motifs: a nucleotide-binding site (NBS) and a leucine-rich repeat (LRR). The proteins encoded by these NBS–LRR genes are thought to act as receptors that can distinguish avirulence factors of pathogens that trigger plant defence responses.[2] The NBS domain was proposed to be crucial for adenosine triphosphate (ATP)-binding and the overall function of the *R-*gene product. The LRR domain is responsible mainly for the genetic specificity of the interaction between the *R-*gene product and the avirulence factors.[3–6] The NBS domains of all characterized *R* genes contain several highly conserved motifs: a P-loop, kinase-2, kinase-3a, and Gly-Leu-Pro-Leu (GLPL) domains.[7] The P-loop and kinase-2 motif are thought to be ATP- and guanosine triphosphate (GTP)-binding sites.[8,9] When a host plant is infected by a pathogen, the *R-*gene can distinguish the avirulence factors of the pathogen and trigger a local hypersensitive response, which leads to programmed death in the plant cells close to the site of infection.

Previous studies have demonstrated that the NBS and LRR domains of the proteins encoded by the resistance gene analogues (RGAs) isolated from different plant species have highly homologous amino acid sequences. A polymerase chain reaction (PCR)-based approach has been developed to clone NBS–LRR-containing *R*-genes on the basis of degenerate primers designed from the conserved regions of the NBS domain. This method has been applied successfully in many species, such as potato,[10] soybean,[11] maize,[12] sunflower,[13] lettuce,[14] *Brassica napus*,[15] rice,[16] common bean,[17] citrus,[18] coffee,[19] chickpea,[20] grapevine,[21] apple,[22] wheat,[23] chicory [24] and sorghum.[25] NBS–LRR resistance (*R*) genes have been also used as polymorphic markers to locate disease resistance genes in *Arabidopsis thaliana*,[26] wheat,[27] melon,[28] cowpea,[29] tomato plants (*Lycopersicon esculentum Mill*),[30] common bean,[31] cocoa [32] and cotton.[33]

Mango (*Mangifera indica* L.) is one of the most important fruits trees in sub-tropical regions. Many diseases, such as anthracnose, powdery mildew, blackspot, gummosis, phomopsis citri, etc., can affect mango at different periods of its life-cycle and impair the fruit quality and yield significantly. However, few studies on the resistance mechanisms in mango have been reported to date, especially, on *R* genes. Therefore, the isolation and characterization of *R* gene sequences in mango is very important and can provide the theoretical basis for breeding of disease-resistant varieties of mango. In this study, we successfully isolated 16 RGAs in mango, and these results will help improve varieties of mango in the future.

## Materials and methods

### DNA isolation and PCR amplification

**Figure 1.**
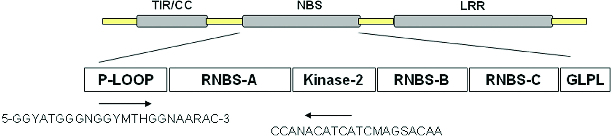
Primer sequences designed based on the P-loop and kinase-2 domain of NBS.

Genomic DNA from the elite mango variety, ‘Jinhuang’, was extracted using the cetyltrimethylammonium bromide (CTAB) method with some modifications.[34] The degenerate primers were designed according to Wang [35]; forward primer: 5′-GGYATGGGNGGYMTHGGNAARAC-3′ and reverse primer: 5′-CCANACATCATCMAGSACAA-3′ (Figure 1). PCR amplification was carried out in a 20 μL reaction containing 50 ng of template DNA, 1 μmol/L primers, 200 μmol/L deoxynucleoside triphosphates (dNTPs), 1×PCR buffer, 2.0 mmol/L MgCl_2_ and 1 U of Taq DNA polymerase (Ferment, USA). PCR conditions included denaturation at 94 °C, then 30 cycles of 94 °C for 30 s, 50 °C for 30 s, 72 °C for 90 s and a final elongation step at 72 °C for 10 min. The amplified PCR products were electrophoresed in 1% (w/v) agrose gels and stained with ethidium bromide. PCR fragments were recovered using the Tiangen DNA gel extraction kit (Tiangen, China).

### Cloning of DNA fragments

Each PCR fragment was cloned into the plasmid, using the pGEM-T Easy Vector system and transformed into *Escherichia coli* strain, following the manufacturer's instructions. Transformed colonies were screened and the presence of an insert was confirmed by colony PCR. Positive clones were sequenced by BGI (China).

### Diversity analysis of cloned DNA fragments using bioinformatics tools

The DNA sequences isolated in this study were compared using software DNASTAR 7.0 and DNAMAN 6.0. Polymorphic values were analysed using DnaSP 4.9 software.[36] The nucleotide sequences of the cloned fragments were translated into amino acid sequences, using the Expert Protein Analysis System Translate Tool ExPASy, a proteomics server. The deduced amino acid sequences were then subjected to motif analyses, using the online version of the CLUSTALW multiple alignment programme of the European Bioinformatics Institute. The amino acid sequences of the 16 RGAs were compared with all protein sequences deposited in GenBank, using BLASTP algorithm.[37] Pair-wise comparisons of RGA sequences at the NBS region were made using the BL2SEQ algorithm.[38]

## Results and discussion

### Isolation of mango *R* gene analogues

**Figure 2.**
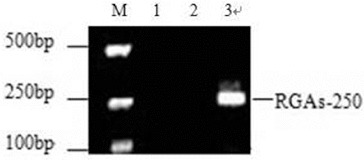
PCR amplification products of NBS RGAs in mango. M: DNA Marker V; Lane 1: blank control; Lane 2: control using water as a template; Lane 3: PCR products of NBS genes amplified with forward and reverse primers for mango.

Using degenerate primers designed based on a conserved motif, we amplified a 250 bp DNA fragment termed RGA-250 from the genomic DNA of the elite mango variety, ‘Jinhuang’ (Figure 2). PCR products of RGA-250 were purified and connected to pGEM-T Easy Vector. Then, 23 positive clones were obtained after antibiotic selection. These 23 positive clones were sequenced, any non-specific sequences were removed and the identical sequences were merged. Finally, a total of 16 sequences, numbered pp-1–16, were obtained. The sequencing results showed that the sequence lengths of pp-1–16 were between 246 bp and 261 bp, and for all of them the amino acid sequences could be deduced. These 16 sequences were submitted to GenBank (accession number: HM446507-22).

The discovery of the conserved motif in the NBS–LRR resistance gene provided a novel approach to clone RGAs, using the PCR-based method. This approach has been successfully applied in different species to investigate resistance analogues and their evolutionary relationship. But research works on isolating RGAs based on degenerate primers have not been documented in mango so far, to the best of our knowledge. In this article, we have isolated and characterized 16 RGAs, using the PCR-based strategy. Sixteen RGAs isolated from genomic DNA of mango can be translated into amino acid sequences, which indicate this method is also practicable for cloning disease resistance genes of mango.

### Gene polymorphism and homologous analysis of mango *R*-gene analogues

**Figure 3.**
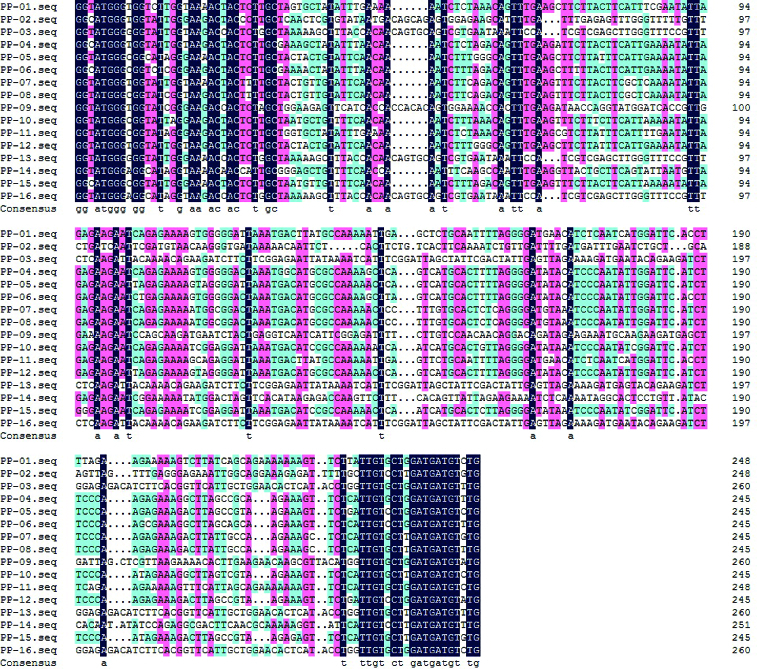
Alignment of NBS-type gene sequences in mango.

**Figure 4.**
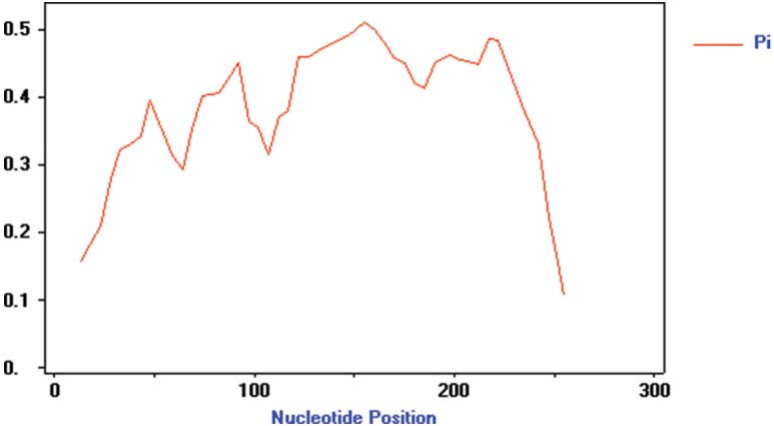
Polymorphism characteristic of NBS–LRR analogues isolated from mango (pp-01–16). The vertical axis represents the nucleotide diversity (*Pi*), and the horizontal axis represents the nucleotide position (bp), the curved line represents the change of *Pi* in different nucleotide positions.

Multiple sequence alignment (DNAMAN 6.0 software) results showed that there were significant differences between the pp-1–16 sequences (Figure 3). Allelic variation among these 16 isolated analogues was analysed using DnaSP4.9 software. There were many types and numbers of variation sites between pp-1–16 (Table 1). Figure 4 demonstrates that the 16 analogues isolated in this study had high level of genetic variation. The total nucleotide diversity index *Pi* was 0.362, the lowest *Pi* value was 0.106 (245–265 bp), and the highest *Pi* value was 0.510 (135–167 bp). In the first and last 20 bp DNA fragment range, the *Pi* values were lower. It is because these regions were located within the P-loop and kinase-2 of NBS conserved regions that the sequence variation was little. In the region between 21 and 254 bp, the *Pi* value was almost above 0.3, and was higher, the closer it was to the neutral position which has the lower intensity of genetic linkage, and is more prone to genetic variation. In short, these results indicate that a variety of resistance genes are available to recognize diverse biotic challenges in the mango genome.

**Table 1:** Variation sites of 16 RGAs sequences in mango.

Type of variation sites	Number of variation sites
Total number of sites (excluding sites with gaps/missing data)	236
Sites with alignment gaps or missing data	29
Invariable (monomorphic) sites	57
Variable (polymorphic) sites	179
Singleton variable sites	17
Parsimony informative sites	162

DNASTAR 7.0 software was used to measure the extent of the homology between the 16 RGA sequences. The extent of the homology between these RGA analogues varied from 44.4% to 98.5%. pp-03 had high homology with pp-13 and pp-16 (average of 98.5%). The lowest identity (44.4%) was found between pp-03 and pp-14. It is possible that pp-03, pp-13 and pp-16 have arisen from the recent duplicate event of a common ancestor gene. However, pp-03 and pp-14 may experience a long period of accumulated divergences since the origin of duplication event. Other RGA analogues showed low to moderate identities ranging from 50% to 80%. These results demonstrate that there was high diversity among the RGAs isolated in the study (Table 2).

**Table 2:** Results from the BL2SEQ algorithm showing the extent of identity between the RGAs isolated in the present study.

	Percent identity															
	pp-01	pp-02	pp-03	pp-04	pp-05	pp-06	pp-07	pp-08	pp-09	pp-10	pp-11	pp-12	pp-13	pp-14	pp-15	pp-16
Divergence																
pp-01		49.0	46.5	83.3	80.9	81.7	79.3	78.0	47.1	78.9	92.8	81.7	46.5	61.7	78.0	46.1
pp-02	86.5		52.8	48.1	47.3	48.5	49.0	48.1	48.8	48.1	48.1	48.5	53.2	47.5	50.2	52.0
pp-03	93.9	74.7		47.5	47.5	45.9	47.1	47.5	59.1	47.5	47.8	49.2	98.5	44.4	46.7	98.5
pp-04	19.0	89.6	90.3		89.0	93.9	85.8	85.8	48.5	87.4	80.5	91.1	46.7	65.4	87.8	46.3
pp-05	22.3	93.1	90.3	12.0		89.0	84.6	84.6	48.1	87.4	80.9	97.6	48.3	64.2	88.2	48.8
pp-06	21.2	88.0	95.9	6.4	12.0		82.1	82.7	48.1	85.0	80.1	89.0	45.9	64.6	85.4	45.0
pp-07	24.6	86.3	91.6	15.9	17.5	20.7		98.4	46.1	85.4	76.8	85.8	47.9	65.8	87.4	46.3
pp-08	26.6	89.1	90.2	16.0	17.6	18.6	1.6		46.5	85.8	77.6	85.4	47.5	65.8	86.6	46.7
pp-09	91.7	90.4	59.5	87.4	88.6	88.5	96.0	94.2		49.4	49.6	49.4	58.4	52.2	48.1	58.8
pp-10	25.0	88.7	90.8	13.9	13.9	16.9	16.4	15.9	84.4		80.1	87.4	48.3	64.6	94.7	47.1
pp-11	7.7	89.6	89.5	22.9	22.4	23.4	28.3	27.1	83.6	23.3		80.1	47.8	63.0	77.6	48.2
pp-12	21.2	88.5	85.0	9.6	2.5	12.0	16.1	16.5	84.5	13.9	23.4		48.3	63.3	87.8	48.3
pp-13	93.7	73.7	1.5	93.0	87.6	95.9	88.9	90.2	61.4	88.0	89.5	87.7		45.2	48.3	96.9
pp-14	54.0	90.5	102.4	46.8	49.0	48.4	45.7	45.7	76.4	48.1	51.3	50.7	99.0		64.6	45.2
pp-15	26.3	82.3	93.2	13.4	12.9	16.5	13.9	14.9	88.7	5.5	26.9	13.5	87.7	48.1		45.9
pp-16	95.3	76.9	1.6	94.6	86.3	99.0	94.5	93.0	60.4	92.3	88.1	87.6	3.1	99.2	96.1	

**Figure 5.**
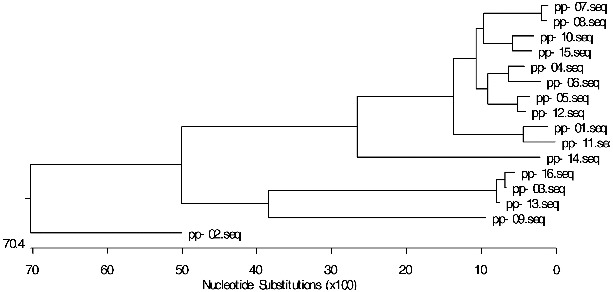
Phylogenetic tree of NBS resistance RGAs in mango.

A phylogenetic tree of the 16 RGAs was constructed by using MegAlign Cluster W program of DNAStar 6.0 software (Figure 5). The 16 mango NBS RGAs were divided into three categories (the threshold value 0.35). The first class includes pp-07, pp-08, pp-10, pp-15, pp-04, pp-06, pp-05, pp-12, pp-01, pp-11 and pp-14. The second class includes pp-16, pp-03, pp-13 and pp-09. The third class includes pp-02 only. It can be seen that these three classes showed a greater degree of genetic disproportionation in the evolution.

### Sequence analysis of conserved motifs

**Figure 6.**
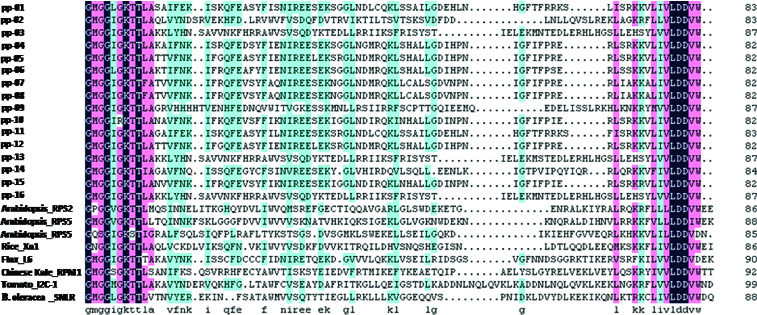
Alignment of amino acid sequences from 16 RGAs of mango, RPS2, RPS5 and PP5 of Arabidopsis, Xa1 of rice, L6 of linen, RPM1 of Chinese Kale and SNLR of sorghum. Identical amino acid sequences are blocked with black.

The amino acid sequences of the 16 RGAs sequences were deduced (Figure 6) and the length of the amino acid sequence was 82 (pp-04, pp-05, pp-06, pp-07, pp-08, pp-10, pp-12 and pp-15), 83 (pp-01, pp-02 and pp-11), 84 (pp-14) and 88 (pp-03, pp-09, pp-13 and pp-16). All 16 amino acid sequences were deduced to contain the P-Loop (GMGGIGKT) and kinase-2 (VLDDVW/D) domain of NBS resistance genes conserved sequence. In addition, kinase-2 (VLDDVW/D) sites were very conservative, all 16 sequences were identical, while the P-Loop (GMGGIGKT) domains were quite conservative, and only glycine mutated to arginine at the sixth point (pp-10). Moreover, in other plants, such as Arabidopsis, the protein sequence of NBS revealed a large variation in RPP2 and RPP5; in the configuration of the conserved domain ‘GMGGIGKTT’, ‘M’ at the second position was mutated to ‘P’ or ‘Q’, at the fifth position ‘I’ and at the seventh position ‘T’ were also mutated to ‘V’ and ‘S’. Furthermore, RPP5, the last point of the conserved domain ‘VLDDVW’ also mutated into ‘D’. These results also showed that the NBS conserved region of mango RGAs was more conservative.

Amino acid sequences of 16 RGAs were compared with other known *R* genes (RPS2, RPS5 and PP5 of Arabidopsis, 12C-1 of tomato, Xa1 of rice, L6 of linen, RPM1 of Chinese Kale and SNLR of sorghum), using the CLUSTAL W multiple alignment program. From the Blast result, the two motif domains of the *R* gene, P-LOOP (GMGGIGKT) and kinase-2 (VLDDVW), kept high conservation among the RGAs, Arabidopsis, rice, tomato, linen, Chinese Kale, and sorghum, which indicated the RGAs isolated in the present investigation may be NBS–LRR resistance genes responsible for specific binding with avirulence factors to promote defence response in mango. PP-02 showed a high identity with I2C-1 of tomato, with a percentage identity of 60%. The identity between the amino acid sequences of PP-01, PP-04, PP-05, PP-06, PP-07, PP-08, PP-10, PP-11, PP-12 and PP-14 varied from 55% to 60%. From Figure 1, the P-loop of the NBS domain seems to be a more conservative region compared with the other sequence in the 16 analogues in nucleotide sequence.

**Figure 7.**
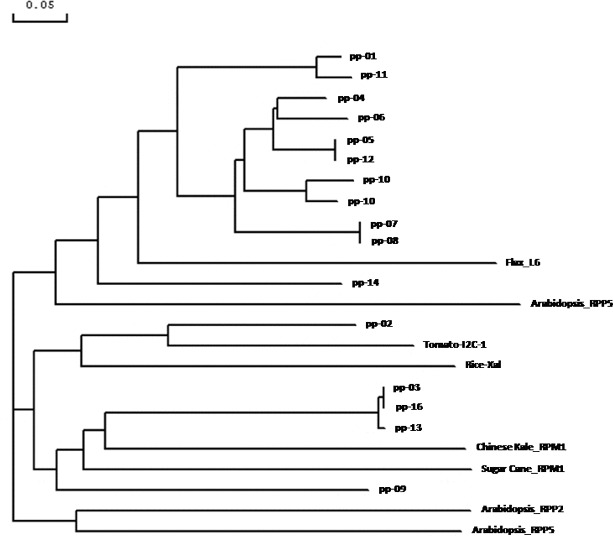
Phylogenetic relationship of amino acid sequences from 16 RGAs isolated in the present study and eight characterized R-genes.

Phylogenetic analysis of RGA sequences with already characterized *R* genes was done by establishing average distance tree, using BLOSUM62 in the Jalview Java alignment editor. We assigned the 24 amino acid sequences into three phylogenetically clustered groups (Figure 7). The largest group contains 11 RGAs (PP-01, PP-11, PP-04, PP-06, PP-05, PP-12, PP-10, PP-15, PP-07, PP-08 and PP-14) and two characterized R-gene (L6 of linen and PP5 of Arabidopsis). The four RGAs, PP-02, PP-03, PP-16 and PP-13, are clustered together with four characterized *R* genes, I2C of tomato, Xa1 of rice, PPM1 of Chinese Kale and RPS5 of Arabidopsis. The other group consisted of RPS2 and RPS5 of Arabidopsis.

RGAs have been isolated based on the PCR-based method using degenerate primers designed according to a conserved motif in a large range of species. Our investigation provided the first presentation of complexity and diversity of *R* NBS–LRR class genes in mango. Some documents have demonstrated rich diversity of RGAs in a given plant genome which has probably been subjected to an unequal cross-over event.[39–41] From our experimental results, RGAs isolated in this investigation also showed a high genetic variation range either in nucleotide sequence or in amino acid sequence except for the P-loop and kinase motif of the NBS domain functioning as an ATP- and GTP-binding site, which may be advantageous to recognize different pathogen avirulence factors. So far, however, it is not known what genetic mechanism is responsible for rich genetic variation of RGAs in plant genomes.

NBS–LRR resistance class genes are a large family with several conserved motifs and have rich copies in plant genomes. For example, there are approximately 150 copies in Arabidopsis and nearly 600 copies in rice.[42,43] The NBS domain, which contains the P-loop, kinase-2, kinase-3a and GLPL, is highly conserved in its protein sequence for the ATP- or GTP-binding and hydrolysis activity.[7] The *R*-class genes can be separated into two clusters according to the presence and absence of a TIR domain at the N-terminus of the protein. The last residues of the NBS domain have been used to predict whether RGAs can be classified into the TIR–NBS family or no-TIR–NBS family, the conservation of tryptophan (W) and aspartic acid (D) are separately characteristics of no-TIR–NBS–LRR protein and TIR–NBS–LRR protein.[44] In our study, 16 RGAs isolated from the genomic DNA of mango were translated into amino acid sequences, sharing good identity with characterized *R-*class genes of L6, PRR5, I2C-1, Xa-1, RPM1, SNLR, RPS2 and RPS5 in the P-loop and kinase-2 domain, which indicates that these RGAs may belong to the R class of NBS–LRR. From our results, the RGAs isolated in this study can be grouped into 2 clusters. But only tryptophan (W) occurred in the last residue of the kinase-2 of the 16 RGAs, which indicates that these RGAs may belong to the non-TIR–NBS–LRR class of the *R* gene family.

## Conclusions

In this study, 16 RGAs isolated from the genomic DNA of mango were translated into amino acid sequences sharing good identity with characterized *R-*class genes of L6, PRR5, I2C-1, Xa-1, RPM1, SNLR, RPS2 and RPS5 in the P-loop and kinase-2 domain, indicating that these RGAs may belong to the R class of NBS–LRR. Our results suggested that the RGAs isolated in this study could be grouped into three clusters. Only tryptophan (W) occurred in the last residue of the kinase-2 of the 16 RGAs, which indicates that these RGAs may belong to the non-TIR–NBS–LRR class of the *R* gene family. 
